# Recurrence Pattern and Complication Rate of Allergic Fungal Sinusitis: A 10-Year Tertiary Center Experience

**DOI:** 10.1155/2020/9546453

**Published:** 2020-12-18

**Authors:** Yazeed Alghonaim, Abdulrhman Alfayez, Riyadh Alhedaithy, Abdullah Alsheikh, Malak Almalki

**Affiliations:** ^1^ORL-H&N Surgery Department, King Abdulaziz Medical City, Riyadh, Saudi Arabia; ^2^ORL-H&N Surgery, King Saud Bin Abdulaziz University for Health Sciences, Riyadh, Saudi Arabia; ^3^ORL-H&N Surgery Department, King Abdulaziz University Hospital, Riyadh, Saudi Arabia; ^4^King Saud Bin Abdulaziz University for Health Sciences, Riyadh, Saudi Arabia

## Abstract

**Background:**

Allergic fungal rhinosinusitis is a noninvasive form of highly recurrent chronic rhinosinusitis. Despite the advancement in medical and surgical strategies, recurrence in AFRS in general poses another challenging problem with reported incidence that eventually can reach more than 60%. Recognition and understanding the pattern of disease recurrence will lead to greater understanding of the disease response in our population.

**Method:**

A retrospective cohort study was performed in King Abdulaziz Medical City in Riyadh, Saudi Arabia. All patients diagnosed with chronic rhinosinusitis and underwent functional endoscopic sinus surgery from the period of January 2006 to December 2016 were reviewed.

**Results:**

28 patients were found to have AFRS based on clinical, radiological, and microscopic examination suggestive of allergic fungal rhinosinusitis. Among these patients, 53% of them were female and 46% were male. The age ranged from 13 to 55 years, with a mean age of 31.57 years. 28.57% of the patients presented with recurrent allergic fungal sinusitis. The duration between the surgery and symptoms recurrence was around one year. Male and female patients had similar recurrence rate (50%). At first visit, 95% of the patients with nonrecurrent disease presented with nasal obstruction compared to 87.5% of the patients with recurrent disease. On the other hand, patients with recurrent disease had more nasal discharge (87.5%), postnasal drip (37.5%), facial pressure/pain (50%), headache (50%), nasal polyposis (87.5%), hypertrophy of inferior turbinate (37.5%), and proptosis (12.5%). Nasal obstruction (87.5%) and nasal polyps (87.5%) were the most common presenting symptoms for the disease recurrence. The pattern of disease recurrence in the previously unilateral disease was 18% ipsilateral and 27% bilateral. For the patients who had bilateral disease formerly, 17% (*n* = 3) of them had recurrent bilateral disease.

**Conclusion:**

Allergic fungal rhinosinusitis is a distinct clinical entity. A high recurrence rate is a pathognomonic feature of the disease, despite all the development in medical and surgical trials. This study demonstrated that recurrence rate is lower in our population. However, more studies with a greater number of patients are needed in the future to clearly recognize the pattern of recurrence in patients with AFRS.

## 1. Introduction

Allergic fungal rhinosinusitis (AFRS) is a noninvasive form of highly recurrent chronic rhinosinusitis. It can be distinguished clinically, histopathologically, and prognostically from the other forms of chronic fungal rhinosinusitis. There are three recognized forms of invasive fungal sinusitis (acute necrotizing, chronic invasive, and granulomatous invasive), in addition to two noninvasive forms (fungal ball and allergic fungal) [1]. Allergic mucin and polyps are the hallmark of the disease [2]. The prevalence of AFRS appears to vary by geographical region. The majority of the reported cases were located in high-temperature regions where the humidity is high relatively [3]. The clinical presentation of AFRS patients includes nasal discharge which generally has a thick, greenish-brown mucoid appearance with a “peanut butter”-like consistency along with green to black rubbery nasal plugs [4].

Diagnosis criteria of AFRS were established by Bent and Kuhn which contain major and minor criteria. Major criteria include type I hypersensitivity, nasal polyposis, characteristics CT scan findings, presence of eosinophilic mucin, and positive fungal smear. The minor criteria include young individuals, coexistence asthma, unilateral predominance, radiographic bone erosion, fungal culture, Charcot–Leyden crystals, and serum eosinophilia [5].

There were multiple research studies done on AFRS locally and worldwide, and few of them questioned the recurrence rate and pattern specifically in the initially involved and noninvolved sinuses. In addition, despite the advancement in medical and surgical strategies, recurrence in AFRS in general poses another challenging problem with reported incidence that eventually can reach more than 60% [6, 7].

Our aim in this research is to shed light on the rate and pattern of recurrence in the sinuses and whether none involved side will be involved later on during the recurrence phase since it is considered as one of the minor criteria for the diagnosis of AFRS.

## 2. Methods

After obtaining the ethical approval, a retrospective cohort study was performed in King Abdulaziz Medical City in Riyadh, Saudi Arabia. Charts of all patients diagnosed with chronic rhinosinusitis (CRS) and underwent functional endoscopic sinus surgery (FESS) from the period of January 2006 to December 2016 were reviewed. Patients fulfilling the Bent and Kuhn criteria for the diagnosis of allergic fungal sinusitis (AFS) were included [5]. Patients without surgical intervention or who were lost follow-up in the given period were excluded. A data collection sheet designed by the researchers included different variables such as demographic information, clinical presentations, site and side of sinuses involvement, course of management, and recurrence rate and pattern. The main outcome variable was the recurrence of allergic fungal sinusitis (AFS). Data analysis was done using SPSS. Descriptive statistics were presented as percentages and frequencies and numerical variables interpreted by means and standard deviations. Inferential statistics were calculated using the chi-square test. A *p* value less than 0.05 was considered significant.

## 3. Results

### 3.1. Patient Characteristics at First Visit

28 patients were found to have AFRS based on clinical, radiological, and microscopic examination suggestive of allergic fungal rhinosinusitis. Among these patients, 53% of them were female and 46% were male. The age ranged from 13 to 55 years, with mean age was 31.57 years. The most common clinical presentation was nasal obstruction (92%), followed by nasal polyps (82%), deviated nasal septum (57%), nasal discharge (50%), and headache (42%). Other clinical presentations include facial pressure/pain (28%), postnasal drip (21%), hypertrophy of inferior turbinate (21%), and proptosis and decreased vision (3%). For the past medical history, 46% of the patients had asthma, 17% had allergic rhinitis, and 7% had aspirin intolerance. 32% of the patients underwent nasal surgical intervention previously. The majority of the patients had FESS (21%), and the remaining had septoplasty (10%).  Table 1 summarizes demographic data, clinical presentation, past medical history, and past surgical history at first visit.

### 3.2. Patient Characteristics in Recurrent vs Nonrecurrent Disease at First Visit

At first visit, 95% of the patients who did not have recurrent disease presented with nasal obstruction compared to 87.5% of the patients with recurrent disease. Nasal discharge was more common in patients with recurrent disease (87.5%) compared to patients with nonrecurrent disease (35%). Patients with recurrent disease had more postnasal drip (37.5%), facial pressure/pain (50%), headache (50%), nasal polyposis (87.5%), hypertrophy of inferior turbinate (37.5%), and proptosis (12.5%). Patients with nonrecurrent disease had more deviated nasal septum (65%) and decreased vision (5%). For the past medical history, patients with recurrent disease had higher incidence of asthma (62.5%) and allergic rhinitis (25%) compared to patients with nonrecurrent disease. On the other hand, aspirin intolerance was only found in patients with nonrecurrent disease (10%). Past nasal surgical intervention was done in 37.5% of the patients with recurrent disease, and 30% of patients with nonrecurrent disease.  Table 2 shows the comparison between the patients who got recurrence and patients who did not in clinical presentation, past medical history, and past surgical history at first visit.

### 3.3. Radiological Characteristics of the First CT Scan

All of the 28 patients had heterogenous opacifications. Maxillary sinus was the most common sinus involved (96%). Anterior ethmoid was involved in 75% of the cases, compared to posterior ethmoid which was evident in 64% of the cases. Additionally, sphenoid sinus was involved in 75% of the cases, and frontal sinus was involved in 71% of the cases. Moreover, 57% of the patients had deviated nasal septum, and 25% had concha bullosa. For the bony defect, 2 patients had infraorbital defect, and only 1 patient had intracranial defect.  Table 3 demonstrates the radiological characteristics of the first CT scan.

### 3.4. Surgical Side and Postoperative Management

Among the 28 patients, 11 patients (39%) underwent unilateral sinus surgery, and 17 patients (60%) underwent bilateral sinus surgery. 21 patients (79%) had mucin intraoperatively. Nasal steroid spray was used postoperatively by 17 patients (57%) while 5 patients (17.86%) used nasal steroid irrigation. In addition, 12 patients (43.86%) used oral steroid as well.  Table 4 demonstrates surgery side and its post-op management results.

### 3.5. Recurrence Rate and Characteristics

28.57% (*n* = 8) of the patients presented with recurrent allergic fungal sinusitis. The duration between the surgery and symptoms recurrence was around one year, but it ranged from 2 months to 2 years. Male and female had similar recurrence rate (50%, *n* = 4). Most of the patients presented with nasal obstruction (87.5%, *n* = 7) and nasal polyps (87.5%, *n* = 7). In addition, half of the patients (50%, *n* = 4) presented with nasal discharge, facial pain/pressure, and headache.

Table 5 summarizes the recurrence rate and characteristics.

### 3.6. Recurrence Side Pattern

The pattern of disease recurrence in the previously unilateral disease was 18% (*n* = 2) ipsilateral and 27% (*n* = 3) bilateral. For the patients who had bilateral disease formerly, 17% (*n* = 3) of them had recurrent bilateral disease. In summary, among the 8 patients with recurrent disease, 2 patients (25%) presented with unilateral disease and 6 patients (75%) presented with bilateral disease.  Table 6 demonstrates disease recurrence pattern side.

## 4. Discussion

In this study, 28 patients were diagnosed to have AFRS depending on clinical, radiological, and microscopic examination suggestive of allergic fungal rhinosinusitis. Our aim was to study the pattern of recurrence of AFRS although we studied multiple aspects including demographic information, clinical presentations, site and side of sinuses involvement, and course of management.

The mean age in our study was 31.57 years with a range of 13–55 years. This is quite similar to a study conducted in India in which the mean age was 28.4 years with a range of 18–48 years [8]. In contrast, in the USA in 2008, the mean age was found to be on the higher side, being 45 years with a range of 18–88 years [9].

The most common signs and symptoms in the first visit were as follows: nasal obstruction 26 (92.86%), nasal polyps 23 (82.14%), and deviated nasal septum 16 (57.14%). This was similar to a study conducted in King Abdulaziz University Hospital, Riyadh, Kingdom of Saudi Arabia, in which the most common signs and symptoms were as follows: nasal obstruction 24 (96%), nasal polyps 22 (88%), and deviated nasal septum 17 (68%) [10].

We assessed the past medical and surgical history in all the patients which showed that bronchial asthma is the most common medical history (46.43%) followed by allergic disorders (17.86%). Past nasal surgeries were found in 32.14% of patients. In contrast, the study which was done in India showed that previous nasal surgeries were seen in 20% of cases followed by bronchial asthma (14.2%) and allergic disorders (11.42%) [8].

Radiological characteristics were as follows: the most common sinuses involved were maxillary (96.43%), anterior ethmoid and sphenoid (75%), frontal (71.43%), and posterior ethmoid (64.29%). We could not find a research study investigating the types of sinuses involved, and most of them compared radiological findings in terms of unilateral vs bilateral involvement. In this research, bilateral involvement was found in 17 (60.71%) vs unilateral 11 (39.29%). In Makkah in a study conducted in 2017, they found bilateral involvement in 36 (69.2%) vs unilateral 16 (30.8%) [11].

We found in our research that 13 (46.43%) patients had recurrence symptoms during their follow-up. However, 8 (28.57%) patients did CT scan which was positive for heterogenous opacification. In contrast to multiple studies, in the eastern province, they found that 54.5% of patients had recurrence, and in Makkah, it was 55% [7, 11]. In addition, we studied the pattern of recurrence and if the previously noninvolved side of the sinuses were involved during the recurrence phase. The incidence of ipsilateral recurrence was 2 (18.18%), and the bilateral recurrence was 3 (27.27%) out of the previously affected unilateral side. 3 (17.65) patients out of bilateral AFS patients who were previously diseased had recurrence. However, in a Makkah study, they found that ipsilateral recurrence 12.5%, contralateral 31.3%, and 61.1% of bilateral AFS patients had recurrence [11].

## 5. Conclusion

A high recurrence rate is a pathognomonic feature of the disease, despite all the development in medical and surgical trials. In addition, more studies with more number of patients are needed in the future to clearly recognize the pattern of recurrence in patients with AFRS.

## Figures and Tables

**Table 1:** Characteristics of patients included in the study.

Demographics		*N*	%
Gender	Male	13	46.43
	Female	15	53.57
Age		13–55	31.57 (mean)
Clinical presentation			
	Nasal obstruction	26	92.86
	Nasal discharge	14	50
	PND	6	21.43
	Facial pressure/pain	8	28.57
	Headache	12	42.86
	Nasal polyp	23	82.14
	DNS	16	57.14
	HIT	6	21.43
	Proptosis	1	3.57
	Decreased vision	1	3.57
Past medical history			
	Asthma	13	46.43
	Allergic rhinitis	5	17.86
	Aspirin intolerance	2	7.14
Past surgical history		9	32.14
	FESS	6	21.43
	Septoplasty	3	10.71

**Table 2:** Comparison between patients with recurrence and patients without recurrence.

		*N* (nonrecurrent patients)	%	*N* (recurrent patients)	%
Clinical presentation		20		8	
	Nasal obstruction	19	95	7	87.5
	Nasal discharge	7	35	7	87.5
	PND	3	15	3	37.5
	Facial pressure/pain	4	20	4	50
	Headache	8	40	4	50
	Nasal polyp	16	80	7	87.5
	DNS	13	65	3	37.5
	HIT	3	15	3	37.5
	Proptosis	0	0	1	12.5
	Decreased vision	1	5	0	0
Past medical history					
	Asthma	8	40	5	62.5
	Allergic rhinitis	3	15	2	25
	Aspirin intolerance	2	10	0	0
Past surgical history		6	30	3	37.5

**Table 3:** Radiological characteristics of the first CT scan.

Radiological characteristics			*N*	%
Heterogeneous opacification			28	100
	Maxillary		27	96.43
		Right	10	35.71
		Left	9	32.14
		Bilateral	8	28.57
	Anterior ethmoid		21	75
		Right	7	25
		Left	7	25
		Bilateral	7	25
	Posterior ethmoid		18	64.29
		Right	7	25
		Left	4	14.29
		Bilateral	7	25
	Frontal		20	71.43
		Right	5	17.86
		Left	7	25
		Bilateral	8	28.57
	Sphenoid		21	75
		Right	6	21.43
		Left	6	21.43
		Bilateral	9	32.14
	Concha bullosa		7	25
		Right	5	17.86
		Left	2	7.14
		Bilateral	0	0
DNS			16	57.14
Bony defect			2	7.14
	Infraorbital		2	7.14
	Intracranial		1	3.57

**Table 4:** Surgery side and post-op management results.

		*N*	%
Surgery			
	Unilateral	11	39.29
	Bilateral	17	60.71
	Mucin	21	79
Post-op management			
	Nasal steroid spray	17	57.14
	Nasal steroid irrigation	5	17.86
	Oral steroid	12	43.86

**Table 5:** Recurrence rate and characteristics.

			*N*	%
Recurrence			8	28.57
	Duration since surgery (month)		2.5–27.6	14.9 (mean)
	Gender	Male	4	50
		Female	4	50
	Clinical presentation			
		Nasal obstruction	7	87.5
		Nasal discharge	4	50
		PND	1	12.5
		Facial pressure/pain	4	50
		Headache	4	50
		Nasal polyp	7	87.5
		DNS	0	0
		HIT	0	0
		Proptosis	0	0
		Decreased vision	0	0
	Radiological recurrence			
		Unilateral	2	25
		Bilateral	6	75
		Uninvolved side previously	3	37.5

**Table 6:** Disease recurrence pattern side.

Previous side involved	Recurrence side	*n*	%
Unilateral		11	39.29
	Ipsilateral	2	18.18
	Bilateral	3	27.27
	Contralateral	0	0
Bilateral		17	60.71
	Bilateral	3	17.65

## Data Availability

The data used to support the findings of this study are included within the article.
